# Attitudes towards disordered eating in the rock climbing community: a digital ethnography

**DOI:** 10.1186/s40337-022-00619-5

**Published:** 2022-07-07

**Authors:** Mattias Strand

**Affiliations:** 1 Centre for Psychiatry Research, Department of Clinical Neuroscience, Karolinska Institutet, and Stockholm Health Care Services, Stockholm County Council, 171 77 Stockholm, Sweden; 2 Transcultural Centre, Northern Stockholm Psychiatry, Stockholm Health Care Services, Stockholm County Council, Solnavägen 4, 113 65 Stockholm, Sweden

**Keywords:** Eating disorders, Anorexia nervosa, Bulimia nervosa, Body image, Sports medicine

## Abstract

**Background:**

Rock climbing is an antigravitational sport in which a low body weight may intuitively seem beneficial. A small number of studies have found an increased prevalence of disordered eating among adolescent and adult climbers. However, to date there has been no qualitative research into the attitudes towards disordered eating and body image in the rock climbing community.

**Methods:**

This explorative study employed a netnographic approach with the aim of understanding how topics related to food, dieting, and disordered eating in the climbing community are addressed in online conversations. Discussion forums on nine major climbing websites as well as three climbing-related forums on the online community Reddit were searched for posts and comments related to the research question. The collected data were then assessed through thematic analysis, generating a number of themes and subthemes.

**Results:**

Five overarching themes, labelled “Is there a problem?”, “Subjective experiences”, “Why and how is weight an issue?”, “The importance of context”, and “What can be done?”, were identified among the forum posts. Most forum users acknowledge that eating disorders are indeed a problem relevant to the climbing community, although a significant minority disagrees. While the assumed benefits of a low weight are clearly a dominant idea among climbers, weight may have become less important over time. Forum users also attest to ways in which climbing may in fact be helpful in fostering a positive body image, such as highlighting performance over aesthetics or emphasizing wholesome community values.

**Conclusions:**

This study demonstrates that the topic of disordered eating and negative body image is far from a blind spot or a “dark secret” within the rock climbing community, as is sometimes claimed. An undue focus on low body weight among climbers must be balanced by proper nutritional advice and healthy role models, not least for young climbers who may feel pressured to lose weight as a quick but short-sighted way to boost performance. Clinicians should be aware of the prevailing ‘weight talk’ in the climbing community and be attentive to negative body image and disordered eating in their patients.

## Plain English summary

This study presents an analysis of content related to eating disorders in a number of online discussion forums dedicated to rock climbing. The findings show that the topic of disordered eating and negative body image is far from a blind spot or a “dark secret” within the climbing community, as is sometimes claimed. There is a rich ongoing conversation among climbers on topics related to eating habits, where most forum users acknowledge that eating disorders are indeed a problem relevant to the climbing community. While the assumed benefits of a low weight are clearly a dominant idea among climbers, weight may have become less important over time as climbing has evolved as a sports discipline. Forum users also attest to ways in which climbing may in fact be helpful in fostering a positive body image, such as highlighting performance over aesthetics or emphasizing wholesome community values. Within the climbing community, an undue focus on low body weight must be balanced by proper nutritional advice and healthy role models, not least for young climbers who may feel pressured to lose weight as a quick but short-sighted way to boost performance.

## Introduction

A heightened risk of developing disordered eating is well-documented in athletes of various disciplines [1, 2]. Disordered eating may arise in the form of an eating disorder, such as anorexia nervosa (AN) or bulimia nervosa (BN), or as a subsyndromal form with the presence of symptoms that do not reach formal diagnostic thresholds. As outlined in the *Diagnostic and Statistical Manual of Mental Disorders, 5th Edition* (DSM-5) [3], AN is characterized by restricted energy intake resulting in a significantly low body weight; an intense fear of weight gain; and a distorted experience of one’s own body weight or shape, an undue influence of body weight or shape on self-image, or a persistent lack of recognition of the seriousness of the current low body weight. BN is characterized by recurrent episodes of binge eating (which involves loss of control eating and consumption of unusually large amounts of food over a brief period of time), unhealthy compensatory behaviors to prevent weight gain (such as vomiting, laxative misuse, or excessive physical exercise), and an overemphasis on weight or shape in self-evaluation. Recent estimates based on a large United States population sample indicate a lifetime prevalence of 0.80% and 0.28% for AN and BN, respectively [4]. However, subthreshold disordered eating in the absence of a full eating disorder is much more common: for example, a study among adolescents showed that 24% of girls and 16% of boys reported some eating disorder symptoms [5].

A number of factors associated with participation in sports, such as striving for perfectionism in athletic performance, pressure from coaches, and a high-performance environment, may expose athletes to an even higher risk of developing disordered eating. In a large study on Norwegian elite athletes involving clinical interviews, 20.1% of female athletes and 7.7% of male athletes fulfilled criteria for AN or BN (including forms currently classified as ‘other specified feeding or eating disorders’ in DSM-5) [6]. Among women, eating disorders were most common in athletes who competed in aesthetic sports such as gymnastics or figure skating (42%), whereas among men, those who competed in antigravitational sports such as high jump or long jump were most often afflicted (22%). High prevalences were also observed for both women and men (30% and 18%, respectively) competing in weight-class disciplines such as boxing or wrestling, which often involves weight cycling in order to qualify for a strategically optimal weight class [7]. Although a low body weight, leanness, and a high strength-to-mass ratio confer a competitive advantage in some sports [2], disordered eating is typically associated with significant medical and psychological consequences that may have a negative impact on athletic performance. Disordered eating can give rise to a broad range of medical complications, such as cardiac dysfunction, gastrointestinal problems, reduced bone mineralization, and serious electrolyte imbalances [8]. What was formerly known as the ‘female athlete triad’—the combination of disordered eating, amenorrhea, and osteoporosis—has been reconceptualized into the broader and more comprehensive concept of ‘Relative Energy Deficiency in Sport’ (abbreviated RED-S) referring to an impaired physiological function in terms of metabolic rate, bone health, immunity, protein synthesis, and cardiovascular health which also affects male athletes [9]. An adequate dietary intake is also of utmost importance in recovery and rehabilitation after sport injuries [10]. Moreover, eating disorders are typically accompanied by psychiatric comorbidity such as mood and anxiety disorders [8] and are associated with substantially elevated mortality rates, partly explained by a markedly increased risk for suicide [11, 12].

Rock climbing has been performed as a sport since the late nineteenth century, evolving from a necessary part of alpinism into a recreational activity in its own right. Various forms of rock climbing exist. In what is nowadays called traditional (or ‘trad’) climbing, climbers place removable gear to protect against falls as they ascend a wall. In contrast, sport climbing—developed during the late 1970s and 1980s—relies on permanent bolts and anchors fixed to the rock for protection. In bouldering, rock formations of a lesser height are climbed without the use of ropes and harnesses. Climbing can be performed outdoors on rock or in indoor climbing gyms on artificial structures. All types of rock climbing require a high level of technique, strength, and endurance; however, taller climbing routes tend to emphasize endurance, whereas bouldering often relies more on power. Much like high jump or long jump, rock climbing may be characterized as an antigravitational sport in which a high strength-to-mass ratio has typically been seen to enhance performance [13, 14]. Several climber memoirs give personal testimony to a widespread culture of disordered eating among elite climbers in the 1980s and 1990s [15, 16]. Furthermore, in the 2021 documentary film *LIGHT*, several professional rock climbers are interviewed about their experiences with eating disorders in an attempt to break “the silence about the sport’s darkest secret” [17].

This anecdotal evidence notwithstanding, there has been little scientific research into disordered eating among rock climbers, even though the sports medicine community is certainly aware of the potential risks [18]. In a 2019 small-scale study on the dietary patterns of adolescent recreational and competitive rock climbers (mean age 14.2), 82% of participants did not meet their target energy intake [19]. Notably, 86% did not meet their target carbohydrate intake and 73% did not meet their target fat intake, whereas a large majority met or exceeded their target protein intake—a pattern that is also characteristic for many individuals with restrictive eating disorders [20]. In a 2020 survey study involving an international sample of 498 adult sport climbers, an overall 8.6% prevalence of disordered eating was observed and 4.2% of respondents reported having been treated for an eating disorder [21]. A higher prevalence of disordered eating was seen among female climbers (16.5%) compared to male climbers (6.3%); in the subgroup of elite and high elite female climbers, the prevalence was 42.9%. To date, however, no qualitative research into the ideas, attitudes, and rationales behind disordered eating behaviors in the climbing community has been published. If eating disorders and body image concerns are indeed a “dark secret”, a blind spot, or at the very least a neglected problem in the climbing scene, a reasonable starting point for exploring attitudes and experiences among climbers—amateur as well as pro-level—is paying attention to what is being said on this topic in the many existing anonymous online discussion forums dedicated to rock climbing.

### Objective

In the present exploratory study, a netnographic thematic analysis approach is employed with the objective of understanding how topics related to food, dieting, and disordered eating in the rock climbing community are addressed in online discussion forums. More specifically, the study aims at providing in-depth insight into the cultural dynamics behind ideas and attitudes concerning climbers’ eating behaviors, athletic performance, body image, and mental health issues over and above the straightforward notion of low weight as an advantage in an antigravitational sport.

## Methods

### Methodological framework and reflexivity

Digital ethnography, also known as online ethnography, involves a wide range of methodological approaches towards researching the social worlds, relationships, and modes of expression in online communities [22]. In terms of data sources, digital ethnography often employs bricolage techniques that make use of discussion forums, comment fields, blogs, images, videos, and other online modes of communication to explore a research question. Similar to all ethnographic research, it typically involves elements of participant observation by which the researcher actively engages with the online community under study, even though this often takes the form of mediated contact with participants rather than direct presence. However, digital ethnography—particularly those specific research practices known as netnography [23]—has increasingly been adopted as a solely observational approach, not least concerning potentially sensitive medical topics where it may often be difficult to negotiate access or recruit informants for a traditional ‘offline’ qualitative study [24]. In the field of eating disorders research, a netnographic approach has been applied in, for example, studies on mukbang viewers [25] or on pro-anorexia communities [26]. Digital ethnography has also been combined with approaches emanating from the fields of human–computer interaction and computer-supported cooperative work, such as in the study of consequences of social media content moderation on the perception of eating disorders [27]. The present study makes use of a non-participatory observational netnographic methodology in the study of the attitudes towards disordered eating in online climbing discussion forums. However, a typical netnographic approach usually involves the assumption that online communities constitute fundamentally different habitats compared to the offline world, governed by their own unique set of social rules; this is not the case in the present study, which adheres to a more conventional digital ethnographic view of online communities as an extension of or a complement to offline realities [22].

In reflecting on my own position as researcher and author vis-à-vis the study topic and the communities under study, multiple roles should be mentioned. First, I have a long experience of working as a consultant psychiatrist in the field of eating disorders, with a special interest in sociocultural factors affecting the development and maintenance of disordered eating and body image issues. Second, I am also an amateur climber with some insight into the climbing community, which may be helpful in understanding the nuances of the topic under study and, not least, the jargon used in the online communities. Third, I am the parent of a 12-year-old child who climbs. I have therefore been involved in children’s climbing classes for a few years and had the opportunity to witness the sport from this perspective too. The online communities included in this study (see below) are thus not new or foreign to me, although I have previously not frequented all of them on a regular basis.

### Data sources, collection, and analysis

Utilizing web traffic analysis tool Alexa Internet, Inc. [28], the nine most popular English-language websites dedicated to climbing with a publicly available discussion forum were identified. In addition to these specialized websites, three major Reddit discussion forums dedicated to climbing were included for analysis. Reddit (reddit.com) is a user-generated discussion website consisting of free-access topical discussion communities known as ‘subreddits’. As of August 2021, Reddit claims to have over 52 million daily active users and is rated as the 19th most visited website globally by Alexa Internet, Inc., with 37.5% of its visitors originating in the United States making it the most popular English-language online community [28].

Data were collected in August and September 2021. No retrospective time limit was applied; the oldest available relevant posts in any of the identified forums turned out to be from 2000. The included discussion forums were searched for posts containing any of the terms “anorexi*”, “bulimi*”, “eat*”, “food” “diet*”, “binge”, “vomit*”, “laxative”, “diuretic”, “weight”, and “body”. Naturally, searching an online discussion forum is very different from searching a medical database and although this list of search terms is certainly not exhaustive given the terminology that might be used in an online conversation, it was assumed that it would suffice to identify whole threads on the topic of disordered eating that could then be inspected more closely for relevant individual posts that may not contain any of these specific words. Identified forum posts that concerned the topic of attitudes towards food, body image, and disordered eating in relation to climbing were copied, omitting author user name information, onto separate data files. Posts that merely discussed general nutritional advice for athletes, the best snacks to bring for climbing sessions etc. were not included. In the case of Reddit, the free online tool Thread Downloader for Reddit (reddit-downloader.tkyanko.me) was used to create pdf files of entire forum threads.

As a complement to this data collection procedure, other related sources were assessed in order to enrich the understanding of the study topic. For example, whenever a forum user made reference to a social media post, a blog entry, a video clip, or a magazine article discussing disordered eating or body image concerns in the climbing community, I would access and read the text/watch the video and consider how it related to the other collected data. These sources typically described professional or semi-professional climbers’ own experiences of and thoughts on restrictive eating and body dissatisfaction; since these individuals can be considered public figures, I did not include their data in the formal analysis and presentation even after word spinning (see below). Furthermore, I would also access and observe a large number of forum threads concerned with other topics than those under study, in order to immerse myself in the online communities and gain a fuller understanding of the whole spectrum of issues of relevance to their members.

The collected data were assessed employing what is sometimes called a codebook thematic analysis approach [29] combining the methodological framework outlined by Braun and Clarke [30] with certain elements of conventional qualitative content analysis [31]. In a highly iterative process, (i) the data was first read and re-read and initial ideas for coding categories and overarching themes were drafted. Next, (ii) those excerpts of text that were relevant to the research questions were coded and labelled according to a data-driven ‘bottom-up’ principle avoiding preconceived ideas about what would matter and not matter to the climbing community. In an initial round of analysis, this yielded 19 separate coding categories covering approximately 2 900 units of information extracted from the collected data. These categories were then (iii) grouped into main themes and subthemes. There were no predefined criteria (e.g., number of informant statements needed) to aid in determining what would constitute a separate theme or subtheme; instead, after the initial analysis had yielded a number of categories, meaningful clusters were identified and developed inductively by analyzing patterns and interrelations within the themes. After that, (iv) the themes were reviewed, revisited, and reworked using mapping techniques in order to achieve a meaningful structure in describing the data; this involved several rounds of fine-tuning in which (v) labelling and re-labelling the themes and categories were important elements in iteratively arriving at a final comprehensive analysis. Finally, (vi) illustrative quotes were chosen (and spun; see below) and the findings were presented and contextualized (see Results and Discussion sections). For all coding, qualitative analysis software NVivo 11 was used.

From an epistemological perspective, this methodology reflects a realist approach to the relationship between language and meaning in the data rather than a constructionist and/or reflexive stance [30]. The aim at this point is to *describe* the attitudes and ideas circulating among climbers in online communities rather than to theorize. Thus, elements of quantitative content analysis—such as providing the reader with the number of posts that express a certain idea—become valuable as yet another layer in the description, even though this does not necessarily imply that the more frequent categories are inherently more important. Not least, arguing based on quantity (“I don’t see a lot of other people in here posting about […]!”, etc.) was very common in the online communities under study, which make it interesting to look at the actual number of posts reflecting certain themes. Also, this approach does not automatically involve the assumption that themes and categories somehow pre-exist in or “emerge from” the data; as touched upon above, reflexivity and interpretation are still important aspects of the research process, even in a descriptive context.

### Preregistration and ethics

The study was preregistered on the Open Science Framework (osf.io/yjbr5). Due to its strictly observational nature, sole reliance on publicly available online data, and non-utilization of any personal data, ethical vetting was neither required nor available in accordance with Swedish Ethical Review Authority regulations. In order to ensure full anonymity, no online user names were collected, analyzed, or presented. The specific online communities under study have also been kept anonymous. Furthermore, in order not to expose the data sources, all quotes have been disguised using the automatic word spinner service WordAi (wordai.com), as suggested by Reagle and Gaur [32], and then further transformed manually. After these measures of anonymization, this study fulfills Bruckman’s criteria for “heavy disguise” in online qualitative research [33]. For an in-depth discussion of research ethics in digital ethnography, see the ethical guidelines of the Association of Internet Researchers [34] as well as the approaches suggested by Bruckman [35].

## Results

Five overarching themes were identified among the forum posts. The first theme, labelled *Is there a problem?*, includes posts that discuss whether or not body image disturbance and disordered eating is indeed a real problem in the rock climbing community. A second theme, *Subjective experiences*, deals with forum users’ own experiences of disordered eating and/or body image issues. A third theme, labelled *Why and how is weight an issue?*, includes posts that discuss specific mechanisms that may either result in negative body image and disordered eating in climbers or that may, in contrast, support the development of positive body image. A fourth theme, labelled *The importance of context*, includes posts that describe how the risk of disordered eating may vary between rock climbing disciplines and contexts. A fifth and final theme, labelled *What can be done?*, involves practical suggestions for how the climbing community ought to respond to the challenge posed by disordered eating. Each of these themes, and their subthemes, are described in more detail below.

### Is there a problem?

Around 520 forum posts discuss whether or not body image disturbance and disordered eating exist as real problems in the rock climbing community (see Table 1). Most of these posts support the idea that disordered eating is indeed prevalent among climbers and that the issue should be taken seriously (approximately 380 references). However, a significant number of forum users (approximately 90 references) take an opposing view: they either do not acknowledge that disordered eating is very widespread in the climbing community or describe the discussion as exaggerated and misdirected. For example, many posts express the view that a dedication to “healthy living” should not be confused with eating pathology or that any occurrence of disordered eating does not stem from climbing per se: “I wouldn’t say that *the climbing community* has a problem with food and dieting. Eating disorders are a major part of the current society in general, so of course you’ll find them among climbers too.” Moreover, in response to claims that some professional competition climbers have appeared to be emaciated, users describe strict adherence to diet regimens as an inherent part of all elite-level athleticism and thus a phenomenon at the margin of the climbing community. Occasionally, this view is also expressed in an attempt to discourage rigid dieting among non-elite climbers: “You should keep in mind that professional climbers are just that: professional. You’re most likely not. If you’re not an elite level climber yourself, whatever these pros are doing to tweak their game is not for you.”

**Table 1:** Examples of forum comments for the theme "Is there a problem?"

Subtheme	Examples of forum comments
1a. Yes, the problem exists	I've visited numerous climbing crags all over Europe and everywhere I notice girls looking sick due to low weight and skipping meals before and after a hard climb. Perhaps some of the people commenting here see this as the standard build for climbers, so that they don't even think of them as being anorexic. They would most likely consider me fat. I believe many people aren't aware of anorexia and how risky it is.
	Me and my friend are actually pretty appalled by the recent metamorphosis of certain elite climbers, in the [two specific nations] teams in particular. This wouldn't be such a major problem if there were systematic attempts to detect this at the professional level (such as BMI limits or blood sampling). Since there aren't, I'm just wondering “why the hell is no one commenting on the fact that these climbers went from healthy looking to skeletons in just a year?”
	I've belonged to several different athletic communities, and I'd say that weight obsession is in fact more common among climbers. This typically leads to unhealthy and potentially hazardous lifestyle changes that might not even bring about any real benefits.
1b. Nah, it's not that big a deal	Do some climbers obsess about weight in an unhealthy way? Sure. Do some climbers even try to become underweight in order to boost performance? Sure. Still, if you add these two groups together, they wouldn't even make up ten percent of the climbing community.
	Where can you find all of those "anorectic" climbers? I never run into them. If you truly were anorectic, you couldn't even muster the strength to climb [an easy route] and performing at pro level would be unthinkable. People who are skinny will always exist, because of genetics and healthy habits I suppose
	A lot of climbers put more energy into warning about weight obsession than into just discussing what is actually optimal for performance. To perform at peak level, you need to adhere to some unhealthy habits whether you like it or not and that's the same in all sports. Just being honest about that and not treating people like they're children would be nice. Let us decide for ourselves what we do with the information.
1c. Disordered eating was more common in the past	The whole idea that eating cauliflower will make you climb harder is an 80s thing. It only makes you sound old and out of touch when you say shit like that.
	Eating disorders aren't fashionable anymore. Even though climbers' bodies differ a lot (for example, compare [two elite climbers]), modern professional climbers are usually bulkier, have more muscles, and look far less unhealthy. It seems to me that "athletic" has replaced "famine-victim skinny". Still, climbers are better now than ever.
	There used to be a rumour in the early 1980s that a top UK climber had anorexia. People were saying that he was so weak from undernourishment that he actually had to be carried to the climbing crag. In those days, climbing was more about endurance than now.

As a part of this theme, a number of posts (approximately 50 references) that do acknowledge a tendency for disordered eating among climbers describe it as a thing of the past. Here, a number of legendary climbers of the 1980s and early 1990s that are rumored to have significantly restricted their diets are recurrently mentioned, with a mix of nostalgia, admiration, and ridicule: “Pink lycra went out of fashion. Nowadays there’s no pressure to stay thin just to look hot in your speedos.” Forum users argue that contemporary climbing emphasizes a greater variety of skills, including power, that make weight a less important issue. Moreover, the rise of climbing-specific sports science and the fact that climbing has evolved from an “ascetic” subculture into a mainstream sport are mentioned as promoting healthier body ideals: “The contemporary climbing scene is just SO much more well-informed, like any other modern-day athletic discipline. Climbers today know that it isn’t smart to starve themselves.”

### Subjective experiences

Forum users’ own experiences of disordered eating and body image issues in relation to climbing are described in approximately 450 posts (see Table 2). This involves personal experiences of disordered eating (approximately 140 references) as well as in friends or relatives (approximately 70 references). A large majority of these accounts describe restrictive eating patterns, although a few mention binge eating and other symptoms of BN. Another subtheme is subjective body image issues that are not necessarily associated with disordered eating (approximately 170 references), such as feeling the pressure to be thinner, hearing others make body-shaming comments, or having other climbers commenting on and idealizing their leanness in an uncomfortable way. Not least, forum users that experience themselves as heavier than others describe how this may make them feel awkward: “I’m usually the heaviest guy on the wall and honestly, it can make me pretty uncomfortable sometimes.” Interestingly, however, in response to comments such as these, others often mention their respect for climbers that do not have a stereotypically athletic body shape: “Whenever there are larger people climbing at my local wall, my only thought is ‘Yay! Go for it!’. I think most people feel that way. It should all be about enjoying ourselves, right?” Furthermore, the image of the climbing crag or gym as an elitist environment is not shared by everyone: “Sure, when you’re a newbie you may feel as if everybody is observing you, and maybe they are because that’s just what climbers do when we’re resting and getting ready for our next attempt. But I can assure you that we watch in a supportive way. We want to see you top that route.”

**Table 2:** Examples of forum comments for the theme "Subjective experiences"

Subtheme	Examples of forum comments
2a. Personal experiences of disordered eating	I lost a few pounds last year, and even though I wish I could say I did it in a healthy way, it actually turned into an eating disorder. For a brief period of time I did climb slightly harder, but after that honeymoon phase I was just tired and freezing and getting injured all the time. I'm glad I turned it around in time
	People say I have the 'perfect' climber body and it makes me so mad. My weight is below 50 kg. Why? Because I ate too little and I never went through puberty as I should have. I still struggle with disordered eating. Your ideal body weight should be a weight that you can maintain and feel good about without restricting your food intake. We need to put a stop to this idea that you climb harder if you weigh less. Increasing the 'strength to weight' ratio by dieting is easy but foolish. What you should be doing is increasing your strength.
	Perhaps some of you will not like what I'm going to say but… You're only 15, you shouldn't be dieting. I was also into team sports and tried to lose weight. It can be done in a healthy way but since climbing is a very competitive sport and team mates aren't always thinking about your well-being, it's SUPER easy to lose control. For me, it led to an eating disorder. So yes, it MAY be helpful, but in the end you should prioritize eating healthy and things will turn out great. You're 15 years old, its ok if your diet isn't perfect. The biggest threat would be to develop anorexia
2b. Friends or relatives had disordered eating	I think the reason why some of us are objecting is that many of us know climbers who just wanted to lose a little weight in order to climb harder, and then it spiraled out of control and they ended up with an eating disorder. It's certainly possible to reach 6% body fat in a controlled way, some athletes do it, but you should be aware of the risks.
	Just watch out. My sister was like you a few years ago–training all the time and being super fuzzy about her diet. She's now in intensive care in [a British] hospital with severe anorexia nervosa, and she has had to drop out of university. At the moment, she can't even walk up a flight of stairs, and climbing anything is out the question. You think you won't lose control but it's so easy to slip.
	I'm worried about my friend going on this climbing trip. He had bulimia last year, now he has anorexia and is severely underweight. He's signed up for an advanced-level climbing course next month and the organizers don't know anything about his disordered eating. My biggest fear is that he will simply collapse on the wall and hurt himself, or worse.
2c. Body image issues	If you already have body image issues, my experience is that climbing can make them worse. I've never felt good about my looks, then I started cycling, and then climbing, two sports where being light is an advantage so that wasn't very helpful. When I become more fit I also become more obsessive, it seems. I look at other people climbing and everyone has like this perfect athletic build… and in contrast, I have love handles flopping over the climbing harness. If I couldn't do a certain move (or succeed in a cycling race) I'd blame my weight and swear that I would lose a couple of pounds more.
	Yes, it's definitely a problem. I was in this group of climbers and the girls would constantly be on social media, commenting on and fat-shaming other climber women. Of course, they did it because they were insecure. I think they looked at climbing like elitist rather than inclusive, and when they felt that they weren't as strong or as popular as others, they would turn to gossiping and criticizing in order to be accepted by their pro-click. To me, it felt like something out of a high school drama and sooo toxic. I made sure to find other climbing friends quick. How come climbing affects people this way when it really should be an inclusive and positive activity?
	Body image ONLY becomes a thing for me when I see all these HOT climbing gear—that clearly doesn't fit me. [Climbing gear brand] especially—I can barely fit into their size L… their clothes are just TINY! Stuff like this makes me wonder If I simply have the "WRONG" body for climbing.
2d. Thin-shaming	Also, I'm thinly built but far from anorexic, and it’s so annoying when other climbers assume that I have some sort of mental disorder and starve myself intentionally!
	I am climber and I am very skinny. I've always been thin: when I was a child, when I didn't climb, when I was into other sports, when it didn't do any sports at all—always tall and super skinny. This was never due to an eating disorder. I've never adhered to any diet. I eat whatever I feel like. I've seen a doctor multiple times, but nothing's wrong with me. I guess it's just in my genes. People often tell me that I climb hard because I am skinny. They'll say that those pro climbers all have anorexia and that's why they're so strong. And sure, that may be true for some of them. Many of them, perhaps. But certainly not all. Some of us just have a fast metabolism by nature and gain strength from training hard and being mindful about what we eat. When you say that this or that strong and skinny climber you see at the crag must have an eating disorder, you're being judgmental and insulting when in reality they just put a lot of hard work in.
	I was once lucky enough to stay with a top-level climber for a few weeks. He would probably be considered very skinny by most and some might even see him and think he's malnourished. Truth is he ate more than I did. Huge enchiladas, fast food, and also lots of healthy food too. I weighed 30 or 40 pounds more than him even though I was only slightly taller, and most would think I'm skinny. Bottom line, there's really no way of telling if someone eats too little unless you actually spend time together. Some people are just gifted with the right genes and don't gain weight.

Notably, a fairly large number of posts (approximately 70 references) mention how others tend to assume that someone who has a slender body shape must be restricting their eating—this is seen as just another, albeit less recognized, form of body shaming. Forum users describe how these comments are often used to belittle their climbing performance; i.e., implying that the sole reason that they succeed in climbing a certain route is their low body weight. Notably, a number of the posts in this subtheme were made in response to a 2008 decision by the Austrian climbing federation to not allow underweight athletes to participate in national competitions in an attempt to discourage disordered eating, a move that many forum users appeared to view as unfair.

### How and why is weight an issue?

Around 1 340 posts discuss specific mechanisms that may either result in negative body image and disordered eating in climbers or that may, in contrast, support the development of positive body image (see Table 3). Notably, the number of posts that underscore the importance of a low weight as a vital aspect of climbing and the number of posts that downplay the impact of body weight are almost equal (approximately 510 and 530 references, respectively). Those that claim that a low weight is clearly advantageous typically refer to the benefits of a high strength-to-mass ratio in gravitational sports, as mentioned in the Introduction section, whereas those that are skeptical about the significance of a low weight emphasize other skills in a climber’s repertoire, such as strength, power, technique, footwork, body positioning, psychological aspects, etc. as more important: “If you think you’ve plateaued, I assure you it’s very unlikely to be because of your weight. […] Me, I still have lots of work to do on my technique, my endurance and my core strength – reducing my weight is not a priority.” These two different stances towards weight can appear to be monolithic and well-established in the climbing community, so that forum users that argue for one of the views may do so in explicit opposition to the rival camp: “As far as I’m concerned….. you shouldn’t pay any attention to these ‘Never mind your weight’ people if you really want to improve and raise your game.” However, it is also not uncommon that both views are expressed within the same post; e.g., a forum user may initially describe how aspects other than weight are certainly the most important in a long-term perspective but then conclude their comment by stating that, all other things equal, striving towards a lower body weight may be an optional short-term tactic in order to boost performance. Moreover, forum users discussing the importance of a high strength-to-mass ratio are often ambivalent about whether it is really wise to try to actively reduce their weight, since this often entails a simultaneous loss of muscle mass.

**Table 3:** Examples of forum comments for the theme "How and why is weight an issue?"

Subtheme	Examples of forum comments
3a. A low weight is clearly beneficial	Light people climb better. I didn't come up with that, it's Sir Newton back in the day:-)
	Body weight may not be the most critical issue, but it sure as hell helps and if you say it doesn’t you're a liar. You can pay more attention to your technique if you take off that 10 kg weight vest.
	Legs are dead weight and so we don't train them. Leg muscles will not help you climb better, so training your legs and gaining muscle weight will only be a disadvantage.
3b. A low weight is not that important	Dropping weight isn't the one and only way of getting better at climbing. As a matter of fact, as long as you become a stronger climber, any additional weight is good. If you're already skinny, this holds true even more. Going thin as a skeleton won’t help you climb [extremely hard routes]. Having enough muscle and technique to master the necessary body positions will. As a 95 kg climber who’s [climbed advanced routes], there is absolutely no reason to say you can’t be heavier and still climb hard.
	Fat people climb better than I do. Thin people climb better than I do. The reason? They are better climbers, period. It has nothing to do with their body weight. It would be much more useful for you to focus on improving your footwork, your flexibility, and doing some [finger training] instead of only obsessing about your weight.
	Gained 5 lbs over Christmas from just eating whatever I wanted. Yesterday I had the best climbing session in a long while. You shouldn't get hung up on your weight. Where you're at mentally and physically is so much more relevant.
3c. Losing weight may have negative consequences	[Elite climber] talked about this. He previously cut weight before competitions but quit doing it. He said it made him weak, low on power and energy. He thinks he does much better in competitions when he makes sure to eat healthy and maintains a healthy weight.
	I agree, why would you want to lose weight? Your energy levels will go down, you'll limit your strength/power, you'll damage your immune system—is it really worth it just to lose a little weight and feel marginally lighter when you climb? Not for me it isn't. I've been climbing hard for many years and every time I tried to cut weight for the sake of performance, all that happened is that I felt weaker.
	My joints are much, much more prone to injuries when I drop to a low weight and body fat percentage.
3d. Balance between losing weight and losing muscle	Contrary to what some say, it is really difficult to lose weight by restricting your calorie intake and not also lose muscle while doing it. And then when you have lost muscle mass, it is going to be a LOT harder to gain it back than what it was to lose it. I am now 9 lbs lighter than I used to be a couple of years ago, when I was at my strongest and could manage one-arm pull-ups on both arms. Now I'm nowhere near being able to do that, and my strength-to-weight ratio is not as good either.
	At this point, I could mostly lose weight in the form of muscle mass. It makes me wonder if it's reasonable to keep on losing weight (I might lose some weight but also lose strength because of muscle atrophy).
	The thing is, a lot of climbers are already fairly skinny but still choose to maintain a deficit (or a sort of down-regulated mode) just because they're afraid to gain weight. And when you're in a deficit, you just don't gain much strength. Your strength can keep on improving year after year for decades, but with weight loss you hit a ceiling–you can only get lighter to a certain point and then there's not much more to gain.
3e. Climbing helps with body image	I've also noticed that when I climb or hike, I concentrate more on the way my body functions and not so much on how it "looks". As a matter of fact, I'm actively involved in introducing outdoor activities in the treatment program of a local eating disorder clinic just because I feel it's so helpful.
	Whenever I see other women climbing, I'm so inspired and impressed by how POWERFUL and cool and awesome these girls are! Actually, I'm sure you look amazing. Might take some time to get used to it though. I just got rid of my favorite shirt because I simply cannot button it around my forearms any longer. Too bad! Still, it's an incredible feeling to be strong and able (who cares if my tiny clothes don't fit anymore) so just keep it up!
	Anyone can become a good climber, no matter what body shape mother nature gave us. That's one of the beautiful things about climbing. You can focus on what works for you and build on that, which is precisely why climbing is so much more than just a sport.

A large number of posts (approximately 190 references) discuss the fact that weight loss may be associated with physiological as well as psychological consequences that ultimately result in an unintended negative impact on climbing performance, such as fatigue, low mood, or increased injury proneness. Furthermore, forum users describe how climbing can actually help foster a positive body image, through a focus on ability and performance rather than aesthetics (approximately 60 references).

### The importance of context

Around 310 posts deal with the notion that the risk of disordered eating may vary between rock climbing disciplines and contexts (see Table 4). First, the idea of a distinct cultural divide between trad climbers and sport climbers can be noted (approximately 50 references; see Introduction section for a description of these disciplines). Mostly, certain forum users that promote trad climbing values tend to describe themselves as no-nonsense, gritty, and “dirtbag” masculine—ideals that they also associate with being able to eat large meals, bringing beer to the crag, etc. Similarly, forum users dedicated to so-called crack climbing and off-width climbing often aspire to an online image of toughness. In contrast, they see sport climbers as self-absorbed, thin-skinned, and bourgeois: “Off-width is for the tough and gritty. Sport climbing is for anorectic sissies and middle schoolers.” When these forum users describe sport climbers, the language is typically sardonic and occasionally misogynistic and homophobic. However, since this presumed animosity has turned into somewhat of an established trope in the forums, it is often difficult to determine if individual posts are sincerely meant or if they are deliberately exaggerated and gimmicky.

**Table 4:** Examples of forum comments for the theme "The importance of context"

Subtheme	Examples of forum comments
4a. Trad climbing is different	your typical trad climber has a beard and a beer belly, brings a six pack to the crag and eats roast dinners. forget about sprouts and vitamin water.
	Anorexic soy boys in tight jeans, shirts off but beanies and "ironic" moustaches on, kombucha-in-a-jar-sipping, vegan-protein-bar-munching mf's getting their asses whooped by routes that my 10 year old daughter uses for warming up
	There are definitely those anorectic guys who climb hard as fuck but if they tried to, say, lift a kettle bell their arms would just snap. Put them on [a glacier route] during the winter and they'll freeze to death.
4b. Weight less important in bouldering	A low body weight has more impact on sport/route climbing than on bouldering. You see that in comp climbing too. Boulder problems usually involve more raw power and getting pumped really isn't that big of an issue.
	Could it be that bouldering has become more popular as a discipline in its own right? Climbing used to be all about being light and having maximum finger strength. Nowadays people just power their way up indoor boulder problems and leanness is not so important.
	I've been climbing for decades in this country and elsewhere and I've always found bouldering very inclusive. Be it [indoor climbing center] in the 1990s or [another indoor climbing centre] today, boulderers come in all shapes and sizes and seem to get along no matter what……… and just enjoy themselves. In my experience, lead walls tend to be less inclusive. A friend of mine was never really comfortable climbing at one of the local “big “ walls even though he's [an advanced climber], but he could never quite put his finger on why he felt that way. Just something in the air I guess.
4c. Weight cycling to improve performance	In my opinion, there's nothing wrong with cutting some weight when a big competition is coming up or when you're trying to climb a route at your limit.
	You should train at a healthy maintenance weight so that you feel alright and have enough calories to build muscle, then cut a few kgs just before you really want to perform well.
	During off-season, I eat chocolate and cheese and whatever and when spring is coming up I just quit. This allows me to train with weight during the winter and then lose it when sport climbing season approaches.
4d. Pressure on young climbers	I find it really disturbing whenever I see parents or coaches who encourage kids to drop weight with the purpose of becoming a better climber. They truly ought to know better. It's so important to create a sound training environment and a healthy outlook on food and diet for young athletes. As adults it is our responsibility to promote this.
	We shouldn't overemphasize winning in youth athletics. When I ended up on the podium in a climbing comp, I immediately felt more appreciated by my team mates and I received more attention from the coaches. Now as an adult I notice this all the time. We're told that winning is what really matters, we're ready to sacrifice in order to keep on winning and to remain relevant. Unfortunately, this may also include compromising our health and putting our bodies at risk.
	Risk assessment and risk minimization is a natural part of climbing, so it seems reasonable to extend that awareness to other areas of our sport besides actually ascending a wall. For me, it is obvious that we should not take cultural influences on body ideals lightly, especially if we see that common approaches to coaching may in fact push kids into eating disorder territory. I have mixed feelings about children in competitive athletics in general, but I do know for certain that we as adults and coaches must protect kids from trauma. It is great to teach kids how to take a fall and try again—dedication and grit will undoubtedly be useful in all areas of life. However, it is not so great to encourage an all or nothing attitude in young climbers.
4e. Remembering reasons for climbing	I agree that climbing outdoors in a fabulous setting with friendly people is, as far as I have experienced, at its core profoundly therapeutic and soothing for body and soul.
	Considering the strong focus on beautiful bodies in climbing media, as well as the transformation of climbing culture from experience-based to performance-based, it is hardly surprising if climbers suffer from body image issues and disordered eating.
	Just enjoy being on the wall. They say the number one climber is the person having the most fun!

Another subtheme reflects the notion that bouldering typically involves more powerful moves, which emphasizes the strength part of the strength-to-weight equation (approximately 40 references).

A fairly large number of posts (approximately 80 references) involve descriptions of some type of weight cycling, e.g., deliberately losing weight before a competition or climbing trip in order to optimize performance. There is also an active discussion about the potentially negative impact of competitive climbing on young athletes, who may be pressured by coaches and parents into going on strict non-age-appropriate diets in order to reduce their weight (approximately 40 references). The importance of top-level adult athletes acting as role models in this regard is also underscored—mostly, however, a perceived lack of such role models is noted: “If my daughters were idolizing *insert famous female pro climber* or if I were coaching teen climbers, I would be pretty worried about the current state of the sport.”

A final subtheme involves posts that discuss the importance of remaining true to what is described as more or less inherent values of climbing, which are seen to run counter to destructive contemporary body ideals (approximately 100 references). Forum users that support this perspective tend to dismiss the idea of climbing as an achievement-oriented sport altogether and instead emphasize aspects such as having fun, sharing experiences with friends, being close to nature, etc. as core values of the (predominantly outdoor) climbing community.

### What can be done?

There are relatively few suggestions in the data for how the climbing community ought to respond to the challenge posed by disordered eating (approximately 17 references; see Table 5). These include discussions about if and how staff at climbing gyms should act when they suspect that someone has an eating disorder, how elite climbers can serve as role models, how climbing competitions could be transformed so as to not unduly reward athletes with a low weight, and how each and every one of us has a responsibility to act in ways that promote healthy body ideals and eating habits in fellow climbers.

**Table 5:** Examples of forum comments for the theme "What can be done?"

Theme	Examples of forum comments
5. What can be done?	I never said that all gym staff must be trained to identify and intervene with clients with eating disorder. What I did say is that staff members should have a general understanding of eating disorders and know who clients can contact if they need help. It could be the [eating disorder patient charity] website, a local GP, a local therapist. The way I see it, it's not intrusive if gym staff ask someone if they have talked to their GP about nutrition. That's what they'd do if a client complained about shoulder pain or something like that, right? Mental health shouldn't be any different.
	I'm worried that someone who might already have problems with self-esteem or whatever would just feel worse if a stranger at the gym approached them and commented on their weight. Of course, if you know them you should absolutely say something, but be aware that once you get involved in helping someone with their mental health problems, you must be prepared to stick with them for the long-term if you truly want to be an ally.
	I would suggest that you engage older climbers—it could be pro climbers or just someone at the gym who's really talented—as role models for the kids. You should frame it as something that they want to do in order to improve their game, rather than just being about diet rules that don't make any sense to them.
	BMI is actually a useful indicator of disordered eating, at least in the lower range. If you're very lean and you worry about wrongfully being called an anorexic, perhaps you should pay attention to your BMI and think about if it's really healthy. Sure, I agree that BMI shouldn't be used on its own to bar someone from competing in climbing… but it can be a good screening tool, obviously. In most cases, I don't think it'll be that difficult to either confirm or discard an anorexia nervosa diagnosis.
	As long as organizers keep allowing these "living skeletons" to take as long as they need to climb a route, the race towards lower weights will continue. If they would instead introduce time limits, make sure that routes favor "powerful" moves and that they aren't too lengthy, climbers will simply have to embrace behaviors that keep them strong and healthy.
	So you've seen someone out there who seems to be on the wrong track?! Give them a hand–show them what climbing should be all about

## Discussion

This netnographic thematic analysis of online rock climbing discussion forums shows that there is a rich ongoing conversation among climbers on topics related to disordered eating and body image. Most of the forum users engaged in these discussions acknowledge that eating disorders are indeed a problem relevant to the climbing community. However, a significant minority expresses the contrary view that this focus on disordered eating and negative body image is misplaced, a fad, or exaggerated. While a formal analysis of trends over time was not performed, it can be noted that forum posts from the early 2000s more often tended to express a positive and idealizing view of striving towards thinness, whereas newer posts more often take a critical stance and highlight other aspects of performance than a high strength-to-mass ratio. This is also reflected in a fair amount of posts that describe eating disorders as a problem mainly characteristic of the 1980s and 1990s climbing scene. Taken together, these findings demonstrate that the topic of disordered eating is far from a blind spot or a “dark secret” within the rock climbing community—in contrast, there is an evident awareness (although certainly not shared by all forum users) of the risks associated with an undue focus on weight in climbing.

Forum users are clearly divided regarding the impact of weight loss on climbing performance. Many view a low body weight as an undeniable benefit in an antigravitational sport, whereas an equally large share of posts emphasize other skills, such as technique, power, and psychological aspects, as far more important. An ambivalence can often be noted in posts on this topic; for instance, a comment may initially describe the effects of weight loss as marginal and highlight other climber qualities, only to conclude by “admitting” that, everything else being equal, this marginal gain may be just what is needed in order to succeed. Generally, however, weight loss is described as a short-term tactic rather than a viable long-term strategy. It is evident that even those forum users that deny the importance of a low weight do so in constant dialogue with the idea of the centrality of weight in climbing as a dominant motive of trope. Those that highlight other aspects as more impactful typically have to argue their position, while those that emphasize weight simply point to “the laws of physics”.

Some forum users, while acknowledging the existence of eating disorders among climbers, display a derogatory attitude towards those that strive for thinness in order to boost performance. Here, a conflict of sorts between trad climbers and sport climbers can be noted, where at least some trad proponents tend to dismiss sport climbers as weight-obsessed, effeminate, and childish. Somewhat ironically, this machismo-influenced “adult” critique is often voiced in a highly juvenile tone involving misogynistic and homophobic language, an overblown praise of beer, meat, and fast food, etc.—although, admittedly, it is often difficult to determine if these comments are sincere or if they merely play or ‘troll’ within an established trope. Notably, it has been argued that rock climbing retains a particularly masculine image and culture that may be excluding to those that do not share those attributes and ideals [36].

As bouldering has evolved from mainly being a way of training for longer routes into a climbing discipline in its own right, boulder problems often tend to center around moves that require a lot of power, such as compression moves. Forum users note that this may diminish the perceived importance on low weight in the strength-to-mass equation—indeed, it is often suggested that it would be more beneficent for a boulderer to build muscle than to try to lose weight. This shift in focus may also have spilled over into sport climbing, since modern climbing routes more often incorporate elements typically associated with bouldering. Notably, the growing focus on highly dynamic parkour-style boulder problems in contemporary competitive bouldering were not reflected in the online discussions of eating behaviors, athletic performance, and body image, which may simply reflect the fact that akin to more traditional bouldering, this style still emphasizes powerful moves.

Many forum users describe how rock climbing can actually be helpful in alleviating the pressure to conform to societal standards of beauty and promoting body acceptance. For some, this is achieved through a distinct focus on performance rather than aesthetics; i.e., what the body can do rather than how it looks. Others, however, altogether dismiss the idea of climbing as necessarily being achievement-oriented and instead point to elements such as enjoying oneself, sharing experiences with friends, being close to nature, etc. as helpful in reducing negative body image. Not least, these aspects are highlighted in relation to the idea of rock climbing as a wholesome and spiritually uplifting outdoor activity rather than a typical competitive sport. At times, however, this sense of community is depicted as being threatened by an emerging negative “soccer mom” mentality in climbing, by which parents pressure kids to perform at the expense of their psychological well-being. Some forum users clearly display nostalgia for a time when climbing was more of an “anarchic” activity and top climbers did not care about competitions, sponsorship deals, and diets—it is, however, unclear exactly when this pristine era took place, since there is simultaneous ridicule of the “Lycra crowd” of decades past.

In any case, climbing is undoubtedly becoming more and more popular and has arguably evolved from somewhat of an adventure-focused outdoor subculture into a “regular” sport so that nowadays, many climbers only ever train indoors on plastic grips in a climbing gym. This could imply that some of the potentially helpful elements of climbing as a wholesome close-to-nature activity are diluted and that problems associated with the highly individualized gym and fitness culture at large [37], such as disordered eating, excessive exercise, and illicit substances, will become more prevalent in the climbing community. On the other hand, as many forum users point out, the establishment of climbing as a contemporary athletic discipline may promote modern evidence-based approaches to training and nutrition, reducing the reliance on outmoded ideas about dietary restriction as a key to success.

### Strengths and limitations

To the best of my knowledge, this is the first qualitative research study that explores the ideas, attitudes, and rationales behind disordered eating behaviors in the climbing community. By employing a digital ethnographic methodology, rich data have been collected and analyzed on a potentially sensitive topic where it could have been difficult to negotiate access or recruit informants for a traditional ‘offline’ qualitative study. Even so, the findings presented here should be interpreted in light of a number of limitations. As always, the question of transferability—i.e., whether or not the findings presented here can be transferred to other settings—arises in qualitative studies. For example, only English-language online discussion forums were included in the study, which may affect transferability to other language spheres; however, it was obvious that English was not always the first language of forum users, even if they use English as a lingua franca of online communication. Also, members of the climbing community that are active in online discussion forums may potentially differ from those that are not in significant aspects, such as age. Furthermore, since this was a strictly observational study, it was not possible to ask follow-up questions or clarify the meaning of posted statements. For instance, it cannot be determined if the focus on “healthy living” described in a large number of posts was indeed healthy or if, in actuality, it represented disordered eating habits—it has indeed been noticed that ‘healthism’ may serve as a socially accepted platform for eating pathology [38].

The search strategy outlined in the Methods section most probably resulted in missed forum posts that could have been relevant to the study (e.g., posts in which bulimia was misspelled as ‘bulemia’). However, it should be noted that the aim of a qualitative study such as this is not necessarily to identify and record every single utterance on a topic, but to collect enough data to ensure data saturation—i.e., that no new topical categories emerge when new data are added. The fact that posts with various misspelled terms were indeed identified during data collection suggests that the strategy of locating entire forum threads that could then be subjected to closer scrutiny worked well. Moreover, data saturation was achieved around halfway through the analysis, indicating that there were no important themes or subthemes that would have been discovered through expansion of the data set.

## Conclusions

In sum, this netnographic thematic analysis of online rock climbing discussion forums demonstrates that the topic of disordered eating and negative body image is far from a blind spot or a “dark secret” within the rock climbing community, as is sometimes claimed. The data presented here reflect rich ongoing conversations among climbers on topics related to eating habits, where most forum users acknowledge that eating disorders are indeed a problem relevant to the climbing community. Forum users are divided regarding the impact of weight loss on climbing performance. While the assumed benefits of a low weight are clearly a dominant idea in the field, there are indications that weight may have become less important over time due to a shift in focus towards powerful dynamic moves in route setting and competitive climbing. Forum users also attest to ways in which climbing may in fact be helpful in fostering a positive body image, such as highlighting performance over aesthetics or emphasizing wholesome community values. Within the climbing community, it is vital that an undue focus on low body weight is balanced by proper nutritional advice and healthy role models, not least for young climbers who may feel pressured to lose weight as a quick but short-sighted way to boost performance. For clinicians who see patients that are active rock climbers, knowledge of the prevailing ‘weight talk’ in the climbing community and active attention to negative body image and disordered eating in their patients are instrumental in preventing and/or identifying climbing-related eating pathology.

## Data Availability

The dataset used and analyzed during the current study is available from the corresponding author on reasonable request.

## Abbreviations

AN: Anorexia nervosa
BN: Bulimia nervosa
RED-S: Relative energy deficiency in sport
